# Coping with failures: how emotions, individual traits, expectation-importance and prior experience affect reactions to violated achievement expectations

**DOI:** 10.3389/fpsyg.2025.1506051

**Published:** 2025-03-06

**Authors:** Lara Orphal, Martin Pinquart

**Affiliations:** Developmental Psychology, Department of Psychology, Philipps University of Marburg, Marburg, Germany

**Keywords:** emotions, tolerance of ambiguity, prior experience, importance, expectations, expectation violation, coping

## Abstract

**Background:**

According to the model ViolEx 2.0, individuals cope with expectation violations in three different ways: assimilation (increasing efforts for expectation maintenance), immunization (ignoring or downplaying discrepant information) and accommodation (changing the expectation). Which contextual and personality factors influence expectation maintenance and change is still subject to investigation.

**Objective:**

This study aimed to determine how two academic emotions, confusion (an epistemic emotion) and annoyance (an achievement emotion), as well as Tolerance of Ambiguity (as personality factor), the importance of an expectation and the prior experiences regarding this expectation (situational factors), relate to coping with expectation violations in achievement contexts.

**Methods:**

Vignettes describing achievement expectation violations were presented to an initial sample of 310 participants. The stories varied in importance of an achievement (high, low), prior experience (confirming, disconfirming, no prior experience), and emotional reaction to the achievement failure (confusion, annoyance, no emotional reaction). As outcome measures, participants indicated their subjective likelihood of using three different coping responses to the expectation violation: assimilation, immunization and accommodation. In addition, Tolerance of Ambiguity was assessed using the German version of the Tolerance of Ambiguity Scale.

**Results:**

Overall, annoyance and confusion predicted higher assimilation and lower immunization. Higher Tolerance of Ambiguity predicted higher immunization and lower accommodation, while higher importance of an initially expected outcome resulted in higher assimilation and lower accommodation. Finally, prior expectation confirmation strengthened expectations, resulting in higher assimilation and immunization, and lower accommodation, while disconfirming prior experience was taken into account only for accommodation. The tendency towards accommodation increased with age, and level of assimilation was lower in men than in women.

**Conclusion:**

When trying to stabilize expectations, it is most helpful to frame communication around importance and confirming evidence. The effect of confirming evidence is much greater than that of disconfirming evidence. While two academic emotions, namely confusion and annoyance, increase the intentions to exert efforts and decrease the likelihood of immunization, their effect is also much smaller than the effect of importance. Finally, we conclude that older individuals accommodate more, and higher Tolerance of Ambiguity makes it more likely to maintain expectations despite discrepancies.

## Introduction

### Expectations and expectation violations

The idea that anyone can achieve any remarkable goal with enough effort is as appealing as potentially misleading, and inspired significant research in psychology and related fields (Duckworth, 2016; Dweck, 2017; Kasser and Ryan, 1993; Langer, 1975). Expectations, defined as subjective beliefs about the likelihood of future events (Hoorens, 2012; Oettingen and Mayer, 2002), often persist despite contradictory evidence (Henss and Pinquart, 2023b). This phenomenon is evident across various areas, including achievements, stereotypes, and mental health (Rief et al., 2015). While maintenance of positive expectation can foster healthy optimism and can be critical for success (Burger, 2023), it can be harmful if those expectations are delusional or prevent positive changes from occurring. Overly high achievement objectives adversely affect mental health (Mohr et al., 2023; Wrosch et al., 2007), and in cases of depression and other psychopathologies, persistent negative expectations can impede therapeutic progress (Rief et al., 2015).

How people react to expectation violations and under which conditions they maintain or update their expectations has been studied through different approaches (Pinquart et al., 2021a). Most recently, the ViolEx 2.0 model has been developed to connect research on expectation violations from different fields within psychology, such as clinical, social, educational and developmental psychology, creating an interdisciplinary framework (Panitz et al., 2021). This model formulates different strategies to cope with expectation violations, recognizing that expectation violations can be acknowledged or dismissed. Acknowledgment results either in expectation-update (accommodation) or in behavioral efforts that aim to maintain the expectation despite obstacles (assimilation). For example, after receiving a worse-than-expected grade, an individual may expect lower grades in the future (accommodation) or increase study efforts to maintain and fulfill high expectations (assimilation). Conversely, dismissal of expectation violations (immunization) is achieved by invalidating the contradictory data (data-oriented immunization), or by conceptually making one’s expectation immune to a violation (concept-oriented immunization). For instance, an individual can claim that a low grade at a test does not count as a bad result because the examiner was unfair (data-oriented immunization), or because the grade was still sufficient to pass the course and hence, was a good result (concept-oriented immunization).

The ViolEx 2.0 model hypothesizes that the coping strategy in a given situation depends on individual traits, social influence, the characteristics of the expectation violation, and the internal representation of this experience (Gollwitzer et al., 2018). While existing studies confirmed the influence of some dispositional and situational characteristics (Henss and Pinquart, 2022, 2023a, 2023b; Pinquart et al., 2021b), studies have not yet focused on how academic emotions influence the maintenance or change of achievement expectations subsequent to expectation violations. Our study aims to fill this gap by integrating the role of emotions with well-documented situational determinants of decision-making under uncertainty and coping, such as prior experience and the importance of an outcome. Additionally, we examine Tolerance of Ambiguity (the individual ability to endure confusion, epistemic conflict, and incongruent information) as a critical personality trait influencing the management of uncertainty and related emotions.

### Prior experience

A central question of the ViolEx framework is under which circumstances expectation-disconfirming experience fails to result in expectation-updating. Studies on learning processes have long investigated how experience shapes behavior. For instance, the amount of trials that build an expectation and their consistency are two major determinants of how individuals react to deviations from expectations (Capaldi, 1966; Redish et al., 2007). From a perspective of learning psychology, expectation violations might seem more likely to result in accommodation if prior experience was inconsistent rather than consistently expectation-confirming, and immunization if prior confirming experience was consistent (Redish et al., 2007; Niv, 2019).

Learning from past experiences is also relevant in Lazarus and Folkman’s research on stress, appraisal and coping (Lazarus and Folkman, 1984). In their framework, individuals appraise events as irrelevant, benign-positive, or stressful. They further determine the specific type of stress (harm/loss, threat, challenge) and evaluate their available coping abilities. These evaluations are often based on previous encounters with similar stressors. As subjective appraisals and interpretations determine the perceived stress better than objective indicators, there is variation in how experience affects individual expectations about stress, including estimations of coping capability. This may depend on dispositional and situational factors. Previous research has shown that pleasant and unpleasant, as well as confirming and disconfirming experiences are treated differently. Specifically, many people display optimism and confirmation biases: they tend to take pleasant experience into account, while dismissing unpleasant experience more easily, and preferentially attend to information which confirms their prior beliefs (Garrett et al., 2017; Klayman, 1995; Sharot et al., 2011). Regarding prior experience, we may thus expect expectation-confirming experience to have stronger influence than expectation-disconfirming experience, and even more so if the expected outcome is pleasant. However, as negative experience is especially aversive when violating expectations, we aim to examine confirming and disconfirming prior experience in an exploratory manner.

### Importance

The personal relevance of an event significantly influences stress and coping mechanisms (Lazarus and Folkman, 1984). This has long been explored in cognitive dissonance theory, where the subjective importance of dissonant cognitions determines the intensity of psychological discomfort, along with the ratio of dissonance to consonance in cognitions (Festinger, 1957). Cognitive dissonance research has shown high importance of attitudes to predict less attitude change and greater resistance to contradictory information, notably through trivialization of information, a defensive strategy which is comparable to the ViolEx mechanism of data-oriented immunization (Sherman and Gorkin, 1980; Simon et al., 1995; Zuwerink and Devine, 1996). As the concept of importance was not clearly defined in Festinger’s theory, subsequent approaches to inconsistency management focused on specific types of importance and investigated self-relevance through personal impact, such as on ego-defense, self-consistency or self-verification (Aronson, 1997; Brannon and Gawronski, 2018; Greenwald and Ronis, 1978; Swann, 1990). With regards to coping strategies, Brandtstädter and Greve (1994) hypothesized that high self-relevance of an expectation results in a particular sequence: first immunization (ignoring the discrepancy), then assimilation (additional efforts to maintain and confirm the expectation), and only if expectation violations seem inevitable, accommodation (expectation update) (Brandtstädter and Greve, 1994). The latter is more difficult if the expectation is central to a person’s life views and hardly substitutable. According to Brandtstädter and Renner (1990), the importance of an objective predicts how long individuals endure “disorganization and disorientation” before disengaging from their expectation and accommodating to the evidence (Brandtstädter and Renner, 1990).

Aubert-Teillaud et al. (2023) developed an epistemic inconsistency management model that integrates factors related to prior experience, specifically subjective certainty and reliability of information, with the importance of expectations. Inspired by Pascal’s wager and further aligning with the expected utility hypothesis (Von Neumann and Morgenstern, 1944), Aubert-Teillaud et al. (2023) conceptualize the importance of maintaining or abandoning an expectation as a utility decision concerning the possible consequences for the individual. They propose that individuals make an epistemic bet on the status of contradictory information and weigh it against the potential cost of errors. Along with confirming or disconfirming prior experience, we therefore expect the importance of an expectation to influence the choice of coping strategies. Specifically, we expect high expectation-importance to result in greater expectation maintenance, i.e., more immunization and assimilation, but less accommodation, compared to low expectation-importance.

### Emotions

It has long been assumed that decisions are based on the likelihood and value of potential outcomes, but extensive research has shown that real-world behavior is influenced by cognitive biases and subjective perceptions, in addition to rational considerations (Simon, 1955; Slovic, 1987; Tversky and Kahneman, 1974). Appraisals of likelihood and value are significantly shaped by momentary emotions (DeSteno et al., 2000; Johnson and Tversky, 1983; Zelenski and Larsen, 2002). Even anticipated emotional reactions modify behavior through motivation: believing that one will experience positive or negative emotions in response to an event predicts the intensity of efforts aiming to prevent negative affect (Aspinwall and Taylor, 1997; Bagozzi and Pieters, 1998).

There is substantial reason to believe that emotions play a role in coping with expectation violations, as prediction errors and cognitive conflict are associated with emotions (Barrett, 2017a; Inzlicht et al., 2015; Nerantzaki et al., 2021). While the influence of emotions has not yet been investigated in research on coping with expectation violations, their role in regulating achievement expectations is well-documented in research on goal-setting, goal-adjustment and monitoring of learning. Evaluating personal behavior and its outcomes compared to objectives relies on metacognitive experiences such as feelings of difficulty, confidence, fluency or uncertainty, and emotions (Efklides, 2011; Efklides et al., 2017; Pekrun and Stephens, 2012; Usher and Schunk, 2017). Two types of emotions are especially relevant: (1) epistemic emotions, which are elicited by cognitive tasks and activities involving knowledge (e.g., boredom, confusion, curiosity, or surprise; Pekrun et al., 2017; Trevors et al., 2016), and (2) achievement emotions, which are related to accomplishments and evaluations (e.g., anxiety, pride, relief, or shame; Pekrun, 2006). The same emotion can pertain to both groups. For instance, frustration can be related to the unfulfilled epistemic objective of understanding a problem, and to the failed achievement of solving it. When personal goals are blocked, such emotions are common. Some authors even suggest that emotions specifically express how events relate to personal knowledge, motivations or values (Baumeister et al., 2007; Frijda, 1988; Silvia, 2010). The evaluative monitoring activity that accompanies metacognitive experiences and emotions is crucial to adaptation of strategies and sensible goal-updating (Dent and Koenka, 2016; Hoyle and Dent, 2017; Winne, 2017).

To better understand coping with expectation violations in achievement contexts, we are particularly interested in confusion and annoyance. Confusion is said to be beneficial for learning through increased engagement and deeper cognitive activities (D’Mello et al., 2014; D’Mello and Graesser, 2014; Lehman et al., 2013). However, when left unresolved, confusion turns into frustration and boredom, leading to disengagement (Baker et al., 2010; D’Mello and Graesser, 2011). We thus expect confusion to predict coping strategies that consider the problem in its entirety, such as accommodation and assimilation, but negatively predict more shallow coping like immunization. Annoyance might predict both increased efforts to confirm the expectation, and abandoning an expectation. Since the threshold where frustration turns from increased efforts to giving up is unclear, it is also possible that negative emotions generally predict a greater overall coping score, without favoring a specific strategy. Finally, as emotions are likely related to the value of an outcome (Pekrun, 2006), high expectation-importance may amplify the effect of negative emotions following an expectation violation.

### Tolerance of ambiguity

Ambiguity and uncertainty are assumed to be aversive and metabolically costly (Barrett, 2017b; Proulx et al., 2012). The individual ability to tolerate situations of confusion, epistemic conflict, and incongruent information has been conceptualized as Tolerance of Ambiguity. This personality trait measures the capacity to deal with novelty and complexity, such as unfamiliar, insoluble or contradictory situations, and predicts approach and avoidance strategies when coping with academic stressors (Grenier et al., 2005; Paralkar and Knutson, 2021). Frenkel-Brunswik (1949) defined the construct as “a tendency to resort to black and white solutions, to arrive at premature closure as to evaluative aspects, often at the neglect of reality” (p. 115). Ambiguity can include characteristics such as conflicting evidence and opinions, expert disagreement, insufficient evidence or simply imprecision (Han et al., 2011). The personality trait Need for Cognitive Closure, which measures the desire for certain answers, quick, clear decisions and aversion to ambiguity, correlates with Intolerance of Ambiguity (Webster and Kruglanski, 1994). Research has shown Need for Cognitive Closure to predict both greater accommodation and assimilation (Henss and Pinquart, 2022, 2023b) or assimilation and immunization, indicating a greater need for coping overall (Henss and Pinquart, 2023a). We expect individuals with low Tolerance of Ambiguity to have a greater need for coping when confronted with expectation violations and to react more strongly to situations involving confusion.

## Materials and methods

### Sample characteristics

We obtained an initial sample size of *N* = 310 participants. After exclusion of participants who failed the attention check or indicated non-serious participation, data of *N* = 292 were included in the analyses. The mean age was 35.26 years (*SD* = 15.12). Participants were predominantly female (67%) and from Germany (95%). Our sample was biased toward academic education, with 49% having completed at least a college or university degree and another 35% having a German secondary school diploma that qualifies for university entrance.

### Design and procedure

The study was preregistered at the Open Science Framework (https://osf.io/fqcz3) and conducted online using SoSciSurvey in May 2024. It was approved by the local ethics committee of the Department of Psychology at Philipps University of Marburg in January of 2024 (file number 2023-81 k). We used a convenience sample, recruiting university students using email lists from German universities and non-student participants via advertisements in Facebook groups and the SoSciSurvey Panel. All participants confirmed that they were at least 18 years old and proficient in the German language. They were informed about their rights and provided informed consent.

The first stage of the study involved responding to a block of 18 counterbalanced vignettes, presented in a randomized order. After the vignettes, participants completed the 12-item Tolerance of Ambiguity Scale, German version (Lietz, 2023), to which we added an attention check. They then provided demographical information (age, gender, highest educational degree). On the last page, they answered whether they had completed the study seriously (Yes/No). After completion, participants had the possibility to leave comments in a free-form textbox and received full information about the study’s objectives. The completion time varied between 13 and 20 min.

### Experimental vignettes design

All vignettes described scenarios where achievement expectations were violated. Since positive (better-than-expected) expectation violations are processed differently from negative (aversive) expectation violations (Holroyd and Coles, 2002; Schultz and Dickinson, 2000), we chose to focus exclusively on negative violations, i.e., worse-than-expected achievements. We experimentally manipulated three factors: (1) the importance of the achievement expectation (high/low), (2) the prior history regarding the expectation (previously confirmed/violated /no prior history), and (3) the emotional reaction of the vignette narrator (confused /annoyed/unemotional statement). These manipulations resulted in 2x3x3 (18) combinations, for which we developed 18 background stories with diverse achievement expectations. After combining all stories and predictors to create a fully counterbalanced design, the total pool consisted of 324 vignettes (2x3x3x18). We created 18 blocks of vignettes to ensure that every combination of predictors and every background story were presented to all participants, without repetition of either. Participants were randomly assigned to one block of vignettes, which were presented in a randomized order. This procedure also ensured that every vignette in the 324-story pool was answered by more than one participant.

Our design adhered to recommendations to cover a broad range of scenarios and systematically vary factors influencing respondents’ judgments, enhancing the potential to generalize findings to broader contexts (Aguinis and Bradley, 2014; Auspurg and Hinz, 2015; Baguley et al., 2022). Examples of vignettes are provided on the OSF page for this study (https://osf.io/c862w/).

In each vignette, the narrator first describes their initial achievement expectation (e.g., “*I am learning Spanish and expect to be able to take part in conversations with native speakers.”*), followed by the expectation violation (e.g., “*But then I travel to Latin America and understand very little.”*). Various sentences accompany each scenario to manipulate predictors (e.g., Prior experience: “*Foreign languages are effortless/effortful for me and conversations have always been easy/difficult for me.”;* Importance: *“I hardly ever need it in everyday life/I plan to live here several years”;* Emotion: *“I am confused /annoyed/this does not affect me so much”*). Tendencies of the participants toward assimilation, immunization, and accommodation were measured after each story. A pretest confirmed the realism of the stories and the effectiveness of the manipulations.

### Measures

To assess the ViolEx 2.0 coping mechanisms, participants responded to three self-developed items corresponding to each vignette, presented in a randomized order. For instance, following the previous scenario, the items included: “I will try to get more motivated so that I stick to my expectation” (assimilation), “I am currently too busy, but I am still going to exercize regularly” (immunization), and “The gym is not for me after all; I do not think I will go regularly” (accommodation). Participants rated their likelihood of choosing each action or attitude (“Please indicate how likely you would be to choose the following strategies,” original wording in German) on a scale ranging from 1 (very unlikely) to 5 (very likely).

Tolerance of Ambiguity was assessed using a German version of the Tolerance of Ambiguity Scale (TAS; Lietz, 2023), consisting of 12 items, 7 of which are reverse-coded (e.g., “*What we are used to is always preferable to what is unfamiliar”*).

Demographic variables included gender (female, male, diverse) age in years (free-form response), country (selection from a list), and formal educational attainment (“What is your highest educational degree?” with 9 options ranging from “still at school” to “completed higher education”).

### Statistical analysis

For *a priori* power analyses, we conducted simulations across a range of power by sample size combinations, using the browser application at https://myshinyapps-oo.shinyapps.io/multi-level-logistic-regression/ (Olvera Astivia et al., 2019). Power calculations reliably exceeded 0.8 for simulations involving a minimum of 220 participants. Given the difficulty of calculating power for ordinal mixed model designs, these power estimations were computed for multilevel logistic regression. Power tends to be higher in ordinal than multilevel models, ensuring that this procedure provided conservative estimations.

All analyses were performed using RStudio (Posit Team, 2024; version 2024.4.2.764). For descriptive purposes, we inspected overall coping choice frequencies, and examined associations between assimilation, accommodation, and immunization using Kendall’s *τ* correlations. These analyses were not conducted to test specific hypotheses but rather to provide an overview of the relationships between the constructs. Following recommendations for statistical analyses of vignette studies in psychology (Baguley et al., 2022), we used cumulative link mixed models to assess the effects of predictors (emotions, importance, prior experience, Tolerance of Ambiguity) on coping tendencies (assimilation, immunization, accommodation). As preregistered, we also analyzed interactions between importance and emotions, and between emotions and Tolerance of Ambiguity. We additionally analyzed the effect of overall “emotions” on “coping” as a dependent variable, aggregating coping styles and emotions. The Likert scale responses (5-point scale) were treated as ordinal, given increasing evidence that treating ordinal variables as continuous can lead to inflated error rates, distorted effect sizes and other mistakes (Bürkner and Vuorre, 2019; Liddell and Kruschke, 2018). Our cumulative link mixed models were proportional odds models with a logit link function, implemented using the R package *ordinal* (Christensen, 2023). They included fixed effects for each manipulated predictor, random intercepts for participants and vignettes, and controlled for demographic variables. Importance, emotions, prior experience and gender were dummy-coded using default contrasts in RStudio. The reference levels were low importance, unemotional reactions, no prior experience, and female gender.

Considering debates on the use of maximal or parsimonious mixed models (Barr et al., 2013; Bates et al., 2015), we assessed the necessity of random slopes by exploring the robustness of our findings in models with maximal random slopes, and different combinations of random slopes, many of which encountered convergence issues. We therefore only included random intercepts. Finally, we evaluated the sensitivity of our models to demographic variables, or using effect coding, all of which yielded consistent outcomes. Model comparisons, along with data and scripts, are available on the OSF page of this study (https://osf.io/c862w/).

## Results

### Coping

Across all vignettes, assimilation was the most favored response (33% “very likely,” 12% “very unlikely”), followed by accommodation (20% “very likely,” 17% “very unlikely”). Immunization was the least favored (14% “very likely” and 20% “very unlikely”). Across all vignettes, assimilation demonstrated a distinct distribution pattern characterized with fewest responses in categories 1 and 2, and markedly higher frequencies in categories 4 and 5. In contrast, both accommodation and immunization demonstrated more uniform distributions across all categories.

In most cases, the three coping strategies were negatively correlated to each other. Specifically, assimilation was negatively correlated with accommodation (*τ* = −0.27, *p* < 0.001) while accommodation was negatively correlated with immunization (τ = −0.13, *p* < 0.001). The association of assimilation and immunization was nonsignificant (τ = −0.02, *p =* 0.06), these correlations were confirmed using a linear mixed effects model that accounted for repeated-measurement, yielding consistent results (slightly higher correlations).

Despite sufficient power, the hypothesized two-way interactions between Tolerance of Ambiguity and emotions, and Importance and emotions, were not significant after correcting for multiple testing. As Likelihood Ratio Test statistics equally favored the main effects models against all interaction models (all *p* > 0.78), we used the main effects models for our analyses. The proportional odds assumption was checked for each predictor in all models, and Likelihood Ratio Tests proved cumulative link mixed models to have a better fit than multinomial logistic models or linear mixed models. All analyses and scripts are available on the OSF page for this study (https://osf.io/c862w/).

The coefficients from cumulative link mixed models become meaningful when transformed into odds ratios (OR). These represent the change in the odds of being in a higher category of the outcome variable for a one-unit change in the predictor variable. In our models, odds ratios indicate the likelihood of being in a specific category (1 to 5) of each coping mechanism, versus a lower one. An odds ratio greater than 1 suggests that as the predictor increases, the likelihood of being in a higher category of the coping strategy also increases. Conversely, an odds ratio less than 1 indicates that, as the predictor increases, the likelihood of being in a higher category decreases.

**Figure 1:**
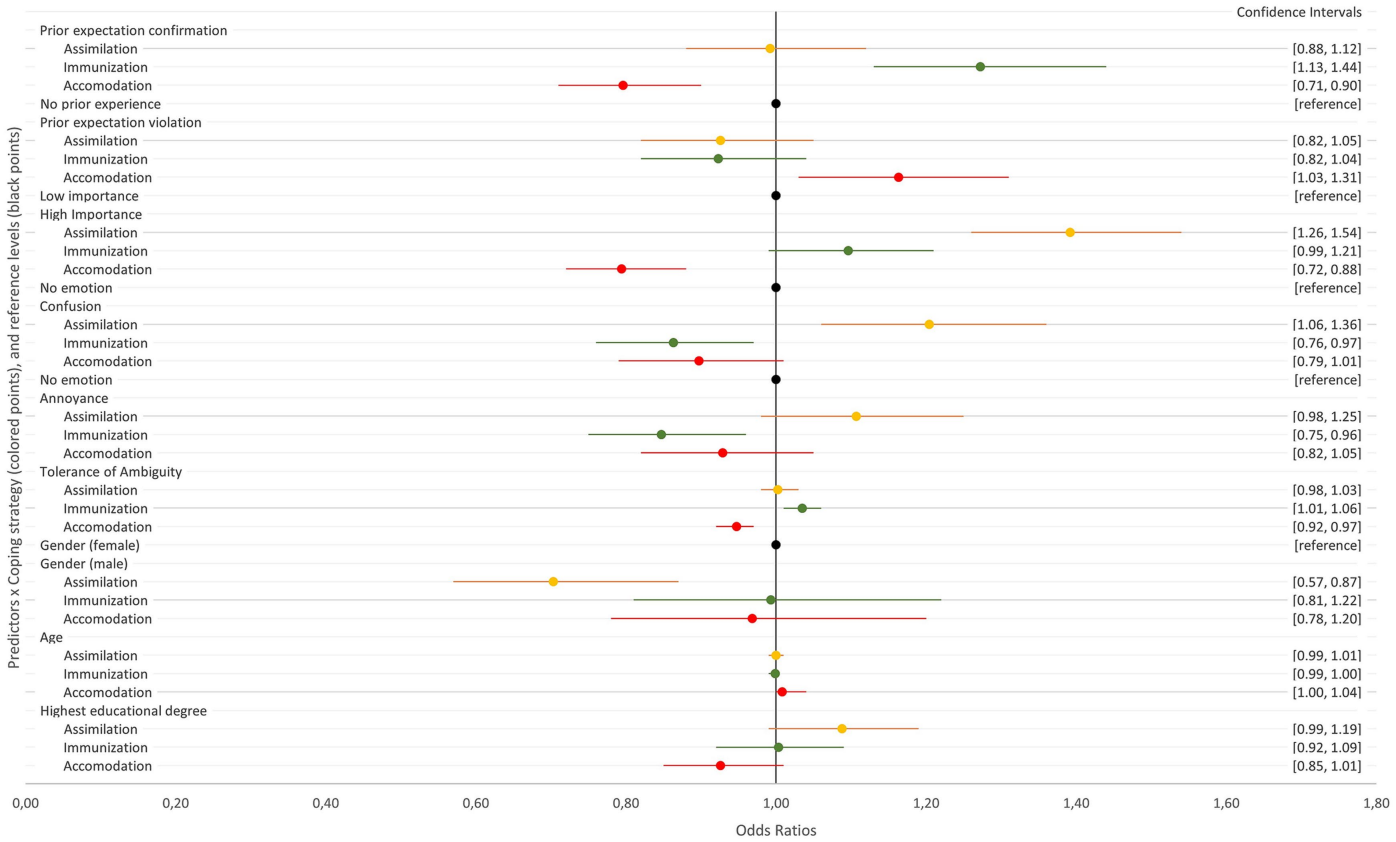
Fixed effects of predictor variables on coping strategies. Estimates are shown as colored points with horizontal bars for 95% confidence intervals, for assimilation (yellow), immunization (green), accommodation (red), and reference levels (black).

Figure 1 allows for a quick visual comparison of the effect sizes (as odds ratios), which are described in detail in the following section. This Figure presents fixed effects estimates for all predictors on assimilation (yellow), immunization (green) and accommodation (red), with the reference levels indicated in black. Each estimate is represented by one colored point, with 95% confidence intervals shown as horizontal bars. The x-axis represents effect sizes, where values to the left of the reference line (1.0) indicate a negative effect and those to the right suggest a positive effect. Only estimates whose confidence intervals do not intersect the reference line indicate statistical significance.

As can be seen in Figure 1, the greatest positive effects are those of high expectation importance on assimilation, followed by prior expectation confirmation on immunization, and confusion on assimilation. To the contrary, the greatest negative statistical effects are those of male gender on assimilation, followed by high expectation importance on accommodation, and prior expectation confirmation on accommodation. As all odds ratios are close to 1, the effects of most predictors on assimilation, immunization, and accommodation are relatively small. For instance, odds ratios such as 1.05 or 0.95 indicate only a 5% increase or decrease in the odds of the outcome. This suggests that while changes in these predictors do influence the response to our vignettes, they only lead to minor shifts in the likelihood of choosing one coping strategy over another.

**Figure 2:**
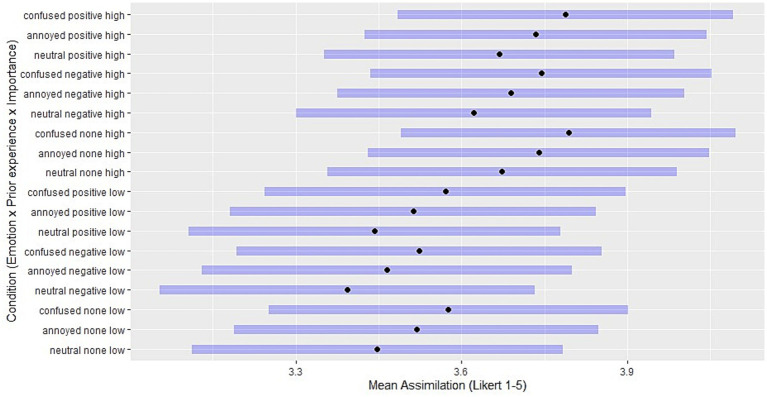
Estimated marginal means: levels of assimilation for each combination of independent variables. Black points indicate the adjusted means for Likert scale assimilation ratings in each manipulation condition, with other covariates controlled. Purple horizontal bars represent 95% confidence intervals. Conditions are a combination of levels of emotion (confused / annoyed / neutral), prior experience (positive, expectation-confirming / negative, disconfirming), and importance (high / low).

**Figure 3:**
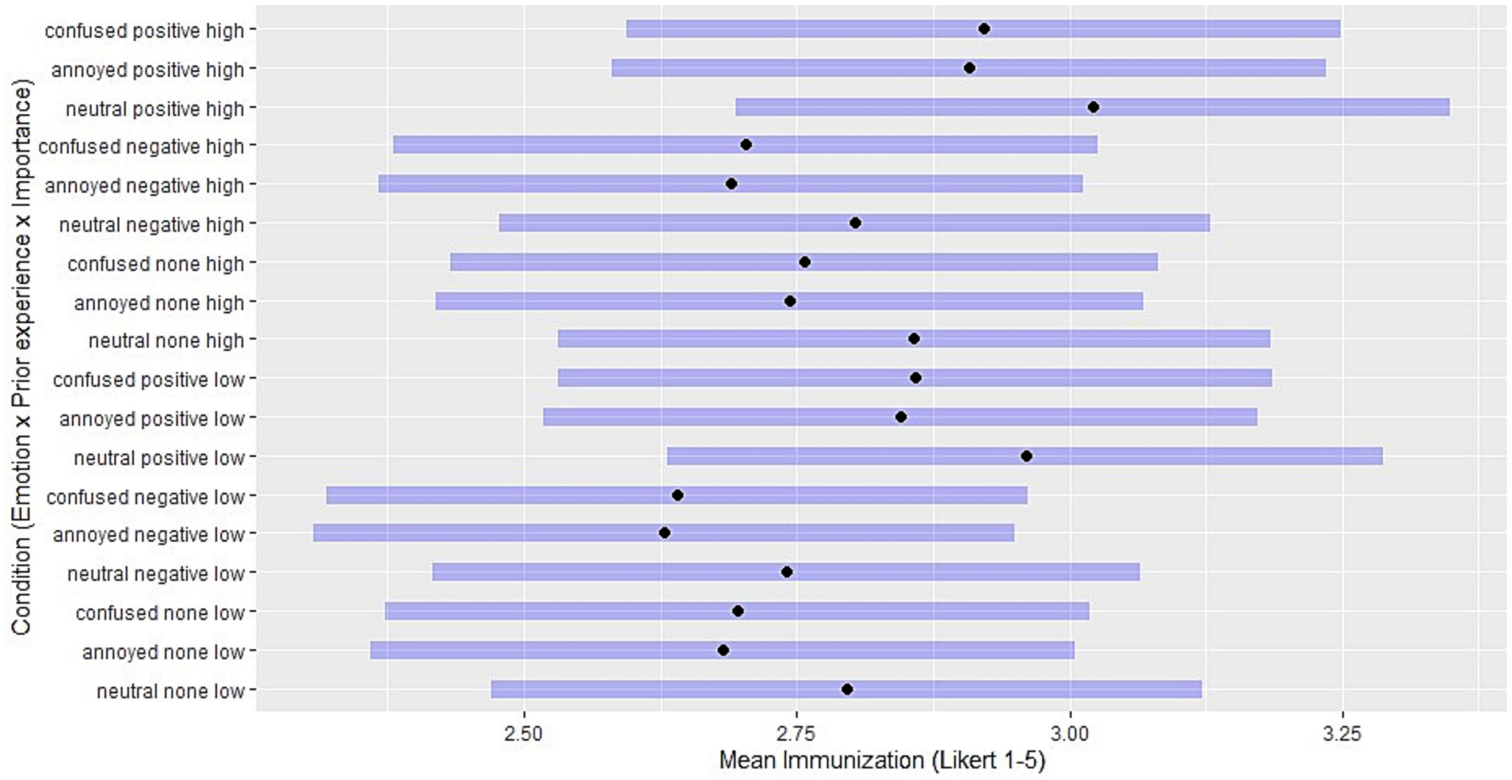
Estimated marginal means: levels of immunization for each combination of independent variables. Black points indicate the adjusted means for Likert scale immunization ratings in each manipulation condition, with other covariates controlled. Purple horizontal bars represent 95% confidence intervals. Conditions are a combination of levels of emotion (confused / annoyed / neutral), prior experience (positive, expectation-confirming / negative, disconfirming), and importance (high/low).

**Figure 4:**
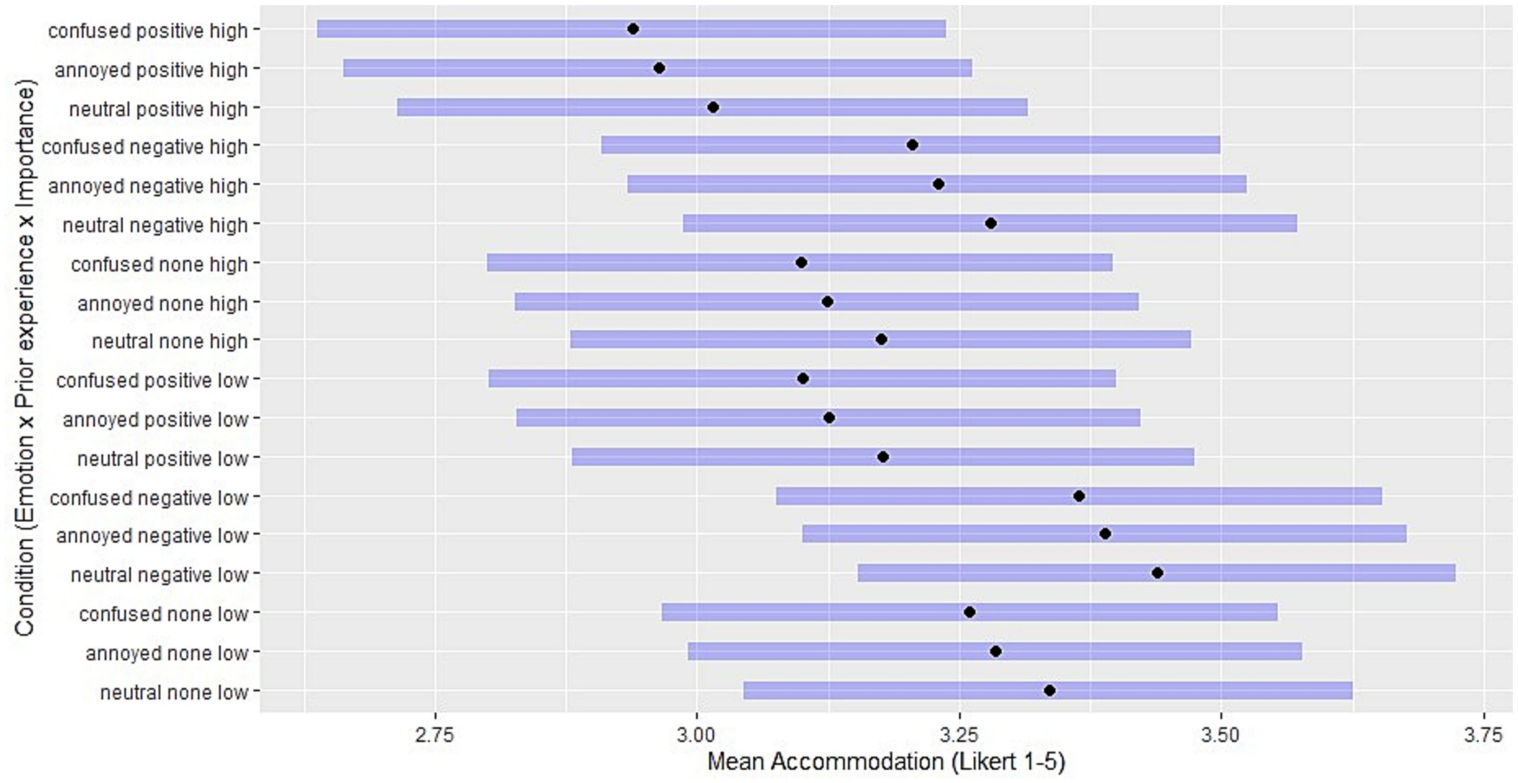
Estimated marginal means: levels of accommodation for each combination of independent variables. Black points indicate the adjusted means for Likert scale accommodation ratings in each manipulation condition, with other covariates controlled. Purple horizontal bars represent 95% confidence intervals. Conditions are a combination of levels of emotion (confused / annoyed / neutral), prior experience (positive, expectation-confirming / negative, disconfirming), and importance (high / low).

For a comparison of mean ratings across manipulation combinations, the Figures 2–4 show the estimated marginal means (EMM) in assimilation, immunization and accommodation, respectively. EMMs represent adjusted means for the ratings in each manipulation condition while controlling for other covariates such as ToA, demographic variables and random effects. For every combination of manipulated predictor levels in the vignettes, a horizontal bar shows the 95% confidence interval.

While the confidence intervals largely overlap, these figures present patterns in how predictor level combinations influence the mean ratings of each coping strategy. For instance, the greatest EMMs in assimilation result from situations with high importance and confused reactions, and the lowest from situations with low importance and a neutral emotion reaction (Figure 2). Conversely, accommodation is greatest when combining negative (disconfirming) prior experience with low expectation importance, and is least chosen when combining positive (confirming) prior experience with high expectation importance (Figure 4). Figure 3 shows that confusion and annoyance result in similarly low levels of immunization, especially under conditions of high expectation importance. Emotionally neutral reactions, on the other hand, are associated with higher levels of immunization, especially when combined with low expectation importance, suggesting that low importance and the corresponding absence of emotional reactions to expectation violations may make it more likely to maintain beliefs despite contradictory experiences.

### Assimilation

Individuals who perceived high importance were 39.2% more likely to choose higher levels of assimilation compared to those perceiving low importance [OR = 1.39, 95% CI (1.26, 1.54), *p* < 0.001]. Additionally, experiencing confusion increased the likelihood of higher assimilation by about 20.4%, as compared to a non-emotional appraisal [OR = 1.20, 95% CI (1.06, 1.36), *p* < 0.01].

Men were 29.7% less likely than women to choose higher levels of assimilation. This finding was statistically significant with an odds ratio of 0.70 [95% CI (0.57, 0.87), *p* < 0.01].

Neither annoyance [OR = 1.11, 95% CI (0.98, 1.25), *p* > 0.1] nor highest educational degree [OR = 1.09, 95% CI (0.99, 1.19), *p =* 0.06] had a significant association with assimilation.

Assimilation was not related to Tolerance of Ambiguity [OR = 1.00, 95% CI (0.98, 1.03), *p =* 0.85], expectation-confirming prior experience [OR = 0.99, 95% CI (0.88, 1.12), *p =* 0.90] or prior experience of expectation violations [OR = 0.93, 95% CI (0.82, 1.05), *p =* 0.23].

The conditional R^2^ indicated that the predictors collectively explained a substantial portion of the variance in assimilation when accounting for both fixed and random effects (R^2^ Cond. = 0.29), although the fixed effects explained only a small portion of the variance (R^2^ Marg. = 0.02).

### Immunization

Immunization was 13.7% less likely in situations described as confusing [OR = 0.86, 95% CI (0.76, 0.97), *p* < 0.05], and 15.3% less likely in situations described as annoying [OR = 0.85, 95% CI (0.75, 0.96), *p* < 0.01]. In contrast, immunization was 27.2% more likely after expectation-confirming prior experience [OR = 1.27, 95% CI (1.13, 1.44), *p* < 0.001], and 3.5% more likely in individuals with higher Tolerance of Ambiguity [OR = 1.04, 95% CI (1.01, 1.06), *p* < 0.01]. High importance had no significant effect on immunization [OR = 1.10, 95% CI (0.99, 1.21), *p =* 0.07].

Immunization was not affected by prior experience of expectation violations [OR = 0.92, 95% CI (0.82, 1.04), *p =* 0.19]. Regarding demographic variables, neither gender [OR = 0.99, 95% CI (0.81, 1.22], *p* > 0.1) nor age [OR = 1.00, 95% CI (0.99, 1.00), *p* > 0.1] or highest educational degree [OR = 1.00, 95% CI (0.92, 1.09), *p* > 0.1] showed significant statistical effects on immunization.

While fixed effects explained only a small portion of the variance in immunization (*R*^2^ Marg. = 0.01), a substantial portion of the variance was explained when accounting for both fixed and random effects (*R*^2^ Cond. = 0.28).

### Accommodation

Accommodation was 20.6% less likely in the case of important expectations [OR = 0.79, 95% CI (0.72, 0.88), *p* < 0.001], 20.4% less likely after expectation-confirming prior experience [OR = 0.80, 95% CI (0.71, 0.90), *p* < 0.001] and 5.3% less likely in individuals with high Tolerance of Ambiguity [OR = 0.95, 95% CI (0.92, 0.97), *p* < 0.001]. It was 16.3% more likely in situations with prior experience of expectation-violations [OR = 1.16, 95% CI (1.03, 1.31), *p* < 0.05], and 0.8% more likely per year of age of the participant [OR = 1.01, 95% CI (1.00, 1.04), *p* < 0.05].

Confusion [10.3% less likely, OR = 0.90, 95% CI (0.79, 1.01), *p =* 0.08], highest educational degree [7.4% less likely, OR = 0.93, 95% CI (0.85, 1.01), *p =* 0.09], annoyance [OR = 0.929, 95% CI (0.82, 1.05), *p =* 0.23] and gender [OR = 0.97, 95% CI (0.78, 1.20), *p =* 0.76] had no significant statistical effects on accommodation.

The conditional *R*^2^ indicated that a substantial portion of the variance in accommodation was explained when accounting for both fixed and random effects (*R*^2^ Cond. = 0.26), but fixed effects explained only a small portion of the variance (*R*^2^ Marg. = 0.02).

### General sum of coping

As preregistered, we intended to investigate whether a generally higher sum of coping strategies used could be predicted by higher emotion and Tolerance of Ambiguity, which required aggregating coping strategies (assimilation, immunization and accommodation) and emotions (annoyance and confusion, vs. non-emotional). We found no evidence confirming our hypothesis. This additional model yielded no statistically significant effect of general emotion [OR = 0.93, 95% CI (0.84, 1.03), *p =* 0.18] or Tolerance of Ambiguity [OR = 0.99, 95% CI (0.97, 1.01), *p =* 0.45] on the aggregated coping tendency score.

## Discussion

This study aimed to investigate how prior experiences, the importance of expectations, emotions, and Tolerance of Ambiguity influence coping strategies when achievement expectations are violated. Our results indicate that each factor plays a significant role in specific coping strategies, and support the ViolEx 2.0 model’s prediction of distinct coping mechanisms, as individuals used strategies differentially based on unique circumstances and personality. We found no indication that emotions or Tolerance of Ambiguity predict generally higher coping when summed-up across strategies, and no confirmation of hypothesized interactions. Ultimately, we provide new insights into situational factors that make individuals likely to immunize, assimilate or accommodate following violated achievement expectations. By understanding these, practitioners can better support individuals in navigating setbacks, and adjust communication to support the adaptive pursuit of objectives. For instance, they can frame situations in ways that specifically encourage persistence, or suggest strategy adjustments when current approaches seem ineffective.

There was no evidence that emotional reactions following expectation violations, or low Tolerance of Ambiguity, predict generally higher coping across strategies. This contrasts with previous research, which found that Need for Cognitive Closure predicts higher accommodation and assimilation (Henss and Pinquart, 2022, 2023b), or higher assimilation and immunization (Henss and Pinquart, 2023a). We found coping strategies to be negatively correlated with each other. Specifically, the coping strategies indicating expectation maintenance were both significantly negatively correlated with expectation change. This indicates that individuals may have to decide whether to maintain or change their expectation, although it may be possible to find a compromise in a few cases. Furthermore, aligning with the Life-Span Theory of Control (Heckhausen and Schulz, 1995), participants in our study generally favored assimilation over other coping strategies. This supports the hypothesis that individuals prioritize primary control, i.e., efforts to change the external world to reach objectives, over secondary control, designating efforts to change oneself to adapt to external circumstances. The latter is only chosen when primary control is deemed unlikely to succeed.

Prior confirming experience predicted higher immunization and lower accommodation, while prior disconfirming experience predicted higher accommodation. As all situations described positive achievement expectations and negative expectation violations, expectation maintenance was favored both by confirmation bias (selectively attending to information which confirms an expectation while ignoring discrepant information) and optimism (maintaining a positive outlook in the presence of obstacles). This aligns with biases reported in the literature (Garrett et al., 2017; Klayman, 1995; Sharot et al., 2011). The importance of expectations tended to have a much greater statistical effect than prior experience overall, and predicted higher assimilation and lower accommodation. This aligns with a long tradition of research on cognitive dissonance and attitude change (Aubert-Teillaud et al., 2023; Sherman and Gorkin, 1980; Simon et al., 1995; Zuwerink and Devine, 1996), suggesting that the importance of an expectation can be a unifying factor across different frameworks.

Confusion and annoyance were found to predict lower immunization and higher assimilation, suggesting that these emotions motivate increased efforts to maintain achievement expectations actively, but make it more difficult or nonsensical to ignore expectation violations. Ignoring or downplaying events may also be more difficult after they already elicited emotions (Gross, 2002). This confirms previous findings that these emotions are associated with increased efforts in academic contexts, although the threshold at which they lead to disengagement may not be reached via hypothetical scenarios (Baker et al., 2010; D’Mello et al., 2014; D’Mello and Graesser, 2011, 2014). Since both emotions were stated explicitly, this result refers mainly to contexts in which individuals are aware of their emotional state or to how they predict reactions when feeling a certain way. Importantly, it indicates that predicting negative emotions following an expectation violation can make individuals exert greater effort to prevent it from happening (again), but is insufficient to change their minds. Thereby, our results connect with research on how anticipated emotional reactions shape motivation and decision-making (Aspinwall and Taylor, 1997; Bagozzi and Pieters, 1998).

Tolerance of Ambiguity was significantly associated with higher immunization, and lower accommodation. These findings indicate a greater cognitive resistance to expectation violations, independently of active behavioral efforts, and suggest that high Tolerance of Ambiguity may reduce the discomfort associated with holding conflicting cognitions, thereby allowing individuals to maintain their original expectations rather than changing them. As individuals with high Tolerance of Ambiguity are better equipped to wait out uncertain situations, it may lead them to favor strategies that preserve existing beliefs. They can possibly take into account counterevidence later, if expectation violations occur again, as described by the concept of “tagging” (Roese and Sherman, 2007). Tolerance of Ambiguity could thus be both beneficial and detrimental in the pursuit of difficult objectives. Accepting a certain degree of contradiction between personal beliefs and counterevidence provides greater resilience when facing obstacles, but enhances the risk to treat occurrences as coincidences, even when alternative strategies are needed.

Age and gender also predicted coping strategies. Female participants assimilated more than their male peers, and accommodation increased with age. In our study, women were willing to make more efforts in order to achieve objectives despite obstacles. One possible explanation could be related to social and cultural factors that encourage adaptability in the face of challenges. Women might be socialized to exert more effort to overcome difficulties (Schlender et al., 2020). Regarding age, it has been hypothesized in past research that accommodation should increase with age as physical or cognitive capacities, and specifically personal control over them, decline (Brandtstädter and Renner, 1990; Heckhausen et al., 2010). Previous research also found growth mindset to decrease with age (Sigmundsson et al., 2022). Older individuals, having accumulated more experience in navigating achievements, may also be less distressed by the prospect of giving up an objective. First, because they have more experience doing so, and second because with age, priorities increasingly focus on close social relationships over other objectives (Carstensen et al., 1999).

### Strengths and limitations

As expectation violations were not experienced in person, but described in hypothetical scenarios, emotions were not felt but considered explicitly. Thus, participants could evaluate the characteristics of expectation-violating situations separately rather than in a phenomenological way (Robinson and Clore, 2002). This may explain why emotions did neither significantly interact with the importance of expectations, nor with Tolerance of Ambiguity. Moreover, all effect sizes were very small, making interactions difficult to detect. While hypothetical scenarios may not evoke the emotional intensity of real-life situations, this design allows distinguishing specific emotions from each other, which is not easily possible in manipulations. Even participants may have difficulties doing so, depending on factors like interoception and emotional granularity (Barrett et al., 2004; Lindquist and Barrett, 2008). While immunization may serve the purpose of suppressing unpleasant emotions early-on via antecedent-focused strategies like reappraisal, downplaying or ignoring information (Gross, 2002), this needs to be tested in future studies, as our experimental design would not be able to capture such relationships. Future research should also investigate these dynamics in real-world settings to validate our findings further. Finally, examining additional emotions and positive expectation violations could provide a more comprehensive understanding of emotions in coping strategies.

## Conclusion

The ViolEx 2.0 model can inform us on expectation updates in achievement situations. Building on this conceptual framework, the present study yielded results that are consistent with related theories, such as models from decision-making, cognitive dissonance, and academic emotions, while discriminating between different coping strategies of expectation maintenance and change. Since ViolEx 2.0 has been used in psychotherapy research and to better understand achievement contexts, therapists, healthcare professionals or educators may be particularly interested in knowing how to adjust communication in order to influence the expectations of a patient, client, or student. Our findings suggest that assimilation is encouraged by highlighting the importance of an expectation and by the academic emotions confusion and annoyance, rather than by prior experience of success or failure. Accommodation is encouraged by diminishing the perceived importance of an expectation, and considering prior disconfirmations. In contrast, the mention of emotional reactions to expectation violations is unlikely to have an impact on expectation update. Immunization is highest with high expectation importance, recalling confirming prior experience, and decreased awareness of emotional reactions to expectation violations. Across all situations, prior experience played a greater role when it was confirming rather than disconfirming. Therefore, professionals are well advised to highlight confirming arguments if they want to strengthen expectations, but may expect less influence from the mention of disconfirming evidence. In sum, when attempting to modify an individual’s expectations, it is recommendable to frame communication around the degree of importance and confirming evidence. In contrast, effects of disconfirming evidence and emotions are much smaller. While emotions may play a role in real-time situations, individuals do not expect them to guide them significantly in their decision to update expectations, as shown in our vignette design. Finally, the tendency toward accommodation increases with age, and individuals with higher Tolerance of Ambiguity will likely persist longer despite discrepancies between beliefs and experience, possibly because they experience less discomfort.

## Data Availability

The datasets presented in this study can be found in online repositories. The names of the repository/repositories and accession number(s) can be found at: Open Science Framework, https://osf.io/c862w/.
